# Using GnRH agonists in combination with hCG in antagonist ART cycles: A SCOPING Review of recent evidences

**DOI:** 10.5935/1518-0557.20230029

**Published:** 2023

**Authors:** Neeta Singh, Aryan Kashyap, Garima Patel, Richa Vatsa

**Affiliations:** 1 Department of Obstetrics and Gynecology, All India Institute of Medical Sciences, New Delhi, India

**Keywords:** trigger, human chorionic gonadotropin, gonadotropin releasing hormone agonist, oocyte maturation

## Abstract

Assisted Reproductive technology encompasses all techniques involving ovarian stimulation to produce high-quality oocytes and manipulation of both oocytes and sperm in vitro to produce embryos for the purpose of reproduction. The final maturation of oocytes induced by a “trigger” is a crucial step with the potential to affect in vitro fertilization outcomes. Human chorionic gonadotropin has traditionally been used as a substitute for luteinizing hormone to induce final oocyte maturation and meiosis. However, this practice may cause a potentially fatal iatrogenic complication known as ovarian hyperstimulation syndrome, which can cause significant morbidity and, in rare cases, death in otherwise healthy women. Thus, gonadotropin releasing hormone agonists have been promoted as a safer alternative for inducing oocyte maturation, albeit at the expense of luteal phase defect. Since then, various combinations of gonadotropin releasing hormone agonists and human chorionic gonadotropin have been tried. This scoping review evaluates these trigger combinations in various types of responders.

## INTRODUCTION

Infertility affects 15% of couples of reproductive age (Gerrits *et al.,* 2017) worldwide and has devastating effects on an individual’s physical, psychological, social, and mental health. Between 1990 and 2017, the burden of infertility increased globally for both genders. Between 1990 and 2017, the age-standardized disability-adjusted life years (DALYs) of female infertility increased by 15.834%, while the DALYs of male infertility increased by 8.843% (Sun *et al*., 2019). Therefore, it is urgent to strengthen assisted reproductive technologies and make them more practical, accessible, and cost-effective for patients with diverse causes of infertility.

Assisted Reproductive Technology (ART) encompasses all techniques involving ovarian stimulation to produce high-quality oocytes and manipulation of both oocyte and sperm in vitro to produce embryos for the purpose of reproduction (Zegers-Hochschild *et al*., 2017). A crucial step of ovarian stimulation protocols in ART is the final maturation of oocytes induced by a “trigger” with the potential to affect in vitro fertilization (IVF) outcomes. Human chorionic gonadotropin (hCG) has traditionally been used as a substitute for luteinizing hormone (LH) to induce final oocyte maturation and meiosis. Even though hCG binds to the same LH/hCG receptor (LHCGR) as LH to produce a response that mimics the mid-cycle LH surge, there is a significant structural difference in their pharmacokinetics and clearance.

Gonen *et al*. (1990) demonstrated that gonadotropin releasing hormone (GnRH) agonists could also be used to stimulate final oocyte maturation in GnRH antagonist cycles by inducing both an LH and follicle stimulating hormone (FSH) surge, which was more physiological than the hCG trigger. The number of oocytes retrieved, the number of mature oocytes (MII), and the number of high-quality embryos were either comparable or favored the GnRH agonist trigger, according to the findings of numerous researchers (Fauser *et al*., 2002; Humaidan *et al*., 2005; Kolibianakis *et al*., 2005; Erb *et al*., 2010). Nonetheless, GnRH agonist trigger resulted in corpus luteum deficiency, luteolysis, and luteal phase deficiency, which resulted in lower pregnancy rates and higher rates of early pregnancy losses (Humaidan *et al*., 2005) due to a significant quantitative reduction in gonadotropins.

Shapiro *et al.* (2008) combined a GnRH agonist with a low dose of hCG in high responders so that a more physiological gonadotropin surge could be obtained, while concomitant administration of low dose hCG would help by supporting the luteal phase. This led to the development of the concept of a ‘dual trigger,’ in which a GnRH agonist is used in conjunction with hCG as a trigger for oocyte maturation. Different studies enrolling patients with diminished ovarian reserve (DOR) (Chern *et al*., 2020; Lin *et al*., 2019; Maged *et al*., 2021) and normal responders (Haas *et al*., 2020; Ali *et al*., 2020) comparing patients receiving dual trigger versus hCG trigger found that the dual trigger group had better fertilization rates, clinical pregnancy rates and live birth rates.

This review attempted to comprehend the physiology of oocyte maturation, the mechanism of hCG and GnRH agonist triggers, the concept of dual trigger, and its application in patients undergoing ART treatments with varying response profiles.

## METHODS

A literature search was done on the following databases: MEDLINE, Google Scholar, Scopus, EMBASE, Global Health, the COCHRANE library, and Web of Science. We examined these data bases from their inception to July 2022 and searched for papers published in the English language. The following Medical Subject Headings (MeSH) and relevant keywords were used in different orders while conducting the literature search: “oocyte maturation,” “trigger,” “human chorionic gonadotropin; hCG,” “gonadotropin-releasing hormone agonist; GnRH agonist,” “double trigger,” and “dual trigger”. The references of all the included studies were also analyzed for studies that were not found in the electronic literature search. A total of 226 articles were found about trigger use in IVF. The study included articles comparing dual trigger with hCG trigger in poor responders, normal responders, and hyper responders. When available, evidence from randomized clinical trials or meta-analyses was given precedence over retrospective studies.

### Physiological foundations of follicle growth and oocyte maturation

Follicle development can be divided into three phases based on their developmental stage and dependence on gonadotropin (Orisaka *et al*., 2009; McGee & Hsueh, 2000):

#### 1. Gonadotropin-independent phase

Approximately two million primordial follicles are present in a female’s ovaries at birth. After birth, primordial follicles remain dormant in the ovaries, but eventually, primordial follicle activation causes a cohort of primordial follicles to transform into primary follicles. Although the precise mechanism of primordial follicle activation is unknown, it has been hypothesized that the absence of inhibitory signals in primordial follicles may be responsible for their activation (Hsueh *et al*., 2015). Recent research indicates that the phosphatidylinositol 3-kinase/protein kinase B (PI3K/Akt) signaling pathway (Hsueh *et al*., 2015; John *et al*., 2008; Zheng *et al*., 2012) and the inhibition of Anti-Müllerian hormone (AMH) (Griesinger *et al*., 2007; Humaidan *et al*., 2010) play a role in the activation of primordial follicles.

#### 2. Gonadotropin-responsive phase

The growth of primordial, primary, and secondary follicles is tightly regulated by intraovarian regulators. In preantral follicles, the gonadotropin responsive phase marks the onset of FSH dependence. This step is crucial in determining the follicular fate - growth versus atresia - beyond the preantral stage, and is responsible for the transition from preantral to antral follicles. This stage also marks the transition from reliance on intraovarian regulators to FSH (Orisaka *et al*., 2009; McGee & Hsueh, 2000). Insulin-like growth factor (IGF), activin, androgens, oocyte-derived factors, bone morphogenetic protein 15 (BMP 15), and connexins are intraovarian regulators that are essential for the acquisition of FSH dependence during the preantral stage (Hsueh *et al*., 2015).

#### 3. Gonadotropin-dependent phase

##### 3.1 FSH-dependent antral follicle growth and LH-dependent maturation

When antral follicles reach a diameter of 2 to 5 mm, circulating FSH and LH regulate their growth. Approximately 5-15 antral follicles begin to grow in response to FSH, but only one dominant follicle is ultimately selected and ovulates (Plant & Zeleznik, 2015).

FSH stimulates the proliferation of granulosa cells of antral follicles by inducing the activity of multiple genes via the activation of numerous intracellular pathways, including the cAMP/PKA pathway, the MAPK pathway, and the PI3K/Akt pathway (Plant & Zeleznik, 2015). In the absence of FSH, granulosa cells undergo apoptosis and follicular atresia develops (Filicori *et al*., 2002). FSH is essential for follicle recruitment and growth, while LH is essential for follicular growth beyond 10 mm in diameter and estradiol (E2) production in the antral phase (Haas *et al*., 2019). During follicular selection, FSH receptor expression is strongly suppressed in the granulosa cells of small antral follicles (2-5 mm), where it is maximally expressed (7-9 mm in diameter). In contrast, during follicle selection, LH receptor expression is significantly increased in granulosa cells (Jeppesen *et al*., 2012). The development of antral follicles is therefore the result of FSH-dependent growth and LH-dependent maturation.

##### 3.2 Determination of the dominant follicle

Selection of the dominant follicle and completion of oocyte maturation requires LH dominance, the ability to produce E2, IGF system activation, and an anti-apoptotic follicular microenvironment (Thierry van Dessel *et al*., 1996). FSH induces LH expression in granulosa cells via multiple intraovarian factors. E2 stimulates the proliferation of FSH and LH receptors and inhibits granulosa cell apoptosis (Plant & Zeleznik, 2015). Follicle selection is a result of the interaction between FSH, E2, and inhibin-B. E2 and inhibin-B, which are secreted by granulosa cells of developing follicles, reduce circulating FSH levels during the mid-follicular phase to stimulate monofollicular ovulation (Hillier, 1994). Only follicles with the highest FSH sensitivity (highest FSH receptor expression) survive and continue to develop as a single dominant follicle even at reduced FSH levels, whereas follicles with low FSH sensitivity undergo apoptosis and follicular atresia as a result of the low FSH milieu (Yding Andersen, 2017).

##### 3.3 Final maturation of the oocyte and ovulation: the LH surge

The pituitary LH surge at the midpoint of the menstrual cycle initiates the final oocyte maturation and follicular rupture. LH surge initiates a series of well-coordinated intracellular pathways that result in particular aspects of oocyte maturation and follicular rupture (Robker *et al*., 2018). Final oocyte maturation involves the resumption of meiosis in order to transition from metaphase I to metaphase II of development. This is the stage in which an oocyte can be fertilized by a spermatozoon (Voronina & Wessel, 2003). The binding of LH to its receptors on granulosa cells induces the activation of multiple intracellular signaling pathways, including protein kinase A, protein kinase C, and Ras (Richards & Ascoli, 2018), which ultimately result in a decrease in intraoocyte cyclic adenosine monophosphate (cAMP) levels, the release of meiosis-promoting factor, and the restarting of meiosis.

### Methods for inducing final oocyte maturation: “Trigger”

Exposure to an LH surrogate is necessary to initiate final oocyte maturation, a crucial step for the success of IVF that results in the retrieval of mature oocytes. hCG or GnRH agonists, commonly referred to as the “triggers” of oocyte maturation, are used in current IVF practice to provide an LH-like exposure (Castillo *et al*., 2014). More than 75% of preovulatory follicles must produce metaphase II oocytes for an oocyte retrieval to be considered “optimal” (Dosouto *et al*., 2019).

### Triggers In ART Cycles

#### 1. Human chorionic gonadotropin

hCG has been the gold standard to induce final oocyte maturation since the inception of IVF. hCG has been utilized as a substitute for the natural mid-cycle LH surge because it is readily available. hCG is a heterodimeric glycoprotein with a high cysteine content and structural similarity to LH, as both proteins share a similar subunit and 85% of the amino acid structure of the subunit (Hershko Klement & Shulman, 2017). Due to their structural similarity, LH and hCG bind to the same LH/hCG receptor (LHCGR). However, not only do their pharmacokinetics and duration of action differ, but they also induce oocyte maturation via distinct molecular mechanisms.

Studies on the pharmacokinetics of hCG (Weissman *et al*., 1996) have concluded that hCG can still be detected in the serum approximately 9-10 days after intramuscular or subcutaneous administration of 10,000 IU hCG. hCG plasma clearance has been shown to be slower, with an initial rapid phase in the first 5-9 hours followed by a slower phase over the next 1-1.3 days (European Recombinant LH Study Group, 2001). hCG has a half-life of 2.32 days, whereas LH has a half-life ranging from 1 to 5 hours (Hershko Klement & Shulman, 2017).

In addition, the molecular mechanisms of action of LH and hCG on LHCGR are distinct. In vitro studies (Riccetti *et al*., 2017; Casarini *et al*., 2012) have demonstrated that LH primarily promotes granulosa cell proliferation, differentiation, and survival by phosphorylating AKT and ERK 1/2, whereas hCG increases cAMP levels, stimulates steroidogenesis, and results in progesterone production.

Ovarian hyperstimulation syndrome (OHSS) is one of the most devastating adverse events associated with hCG trigger, despite its widespread use as an IVF trigger. In the pathogenesis of OHSS, an increased number of granulosa cells due to multifollicular growth and extensive production of vascular endothelial growth factor (VEGF) following luteinization of these cells are implicated. Slower plasma clearance and a longer half-life of hCG increase the luteinizing stimulus to the granulosa cells, thereby playing a crucial role in the development of OHSS (Seyhan *et al*., 2013).

#### 2. GnRH agonists

GnRH agonists cause a more physiological response by inducing an endogenous gonadotropin surge and are a safer option among women at high risk of OHSS compared to hCG (Devroey *et al*., 2011).

Gonen *et al.* (1990) compared the efficacy of a single mid-cycle dose of a GnRH agonist versus hCG for follicular maturation. At midcycle, they randomized 18 IVF cycles to receive either 0.5 mg leuprolide acetate or 5000 IU hCG. Both groups underwent the same ovarian stimulation protocol and cycle monitoring. In their study, they determined that the mean number of oocytes retrieved, embryo quality, and embryo number did not differ between the two groups. Consequently, this study laid the groundwork for the use of GnRH agonists as a trigger in IVF.

GnRH agonists have an advantage over hCG because they induce an endogenous gonadotropin surge of both LH and FSH that closely resembles the natural mid-cycle gonadotropin surge (Humaidan *et al*., 2009). Previous studies have demonstrated that FSH promotes LH receptor formation in the luteinizing granulosa cells, nuclear maturation, and cumulus expansion, although the role of the mid-cycle FSH surge in a natural cycle is still under investigation (Yding Andersen, 2017; 2002; Zelinski-Wooten *et al*., 1995).

A retrospective review (Erb *et al*., 2010) analyzed 32 oocyte donors with adequate ovarian reserve undergoing antagonist downregulated cycles prescribed either leuprolide acetate (n=12) or hCG (n=20) for final oocyte maturation. The authors found that the leuprolide arm produced significantly more total oocytes (23 *vs*. 15), metaphase II oocytes (22 *vs*. 13), embryos (15 *vs*. 10) and cryopreserved embryos (12 *vs*. 6). In a second prospective randomized study, Humaidan *et al.* (2005) randomized 122 normogonadotropic patients following a flexible antagonist protocol to receive either a single subcutaneous bolus of 0.5 mg buserelin (n=55) or 10,000 IU of hCG (n=67). In the GnRH agonist group, significantly more metaphase-II (MII) oocytes were retrieved.

The low incidence of OHSS, which has been attributed to the luteolytic effect of the brief LH surge, is another advantage of GnRH agonists used as a trigger for oocyte maturation (Orvieto, 2015). Itskovitz-Eldor *et al*. (2000) described the use of 0.2mg triptorelin to induce ovulation in eight patients undergoing ovarian stimulation and antagonist down-regulation with recombinant FSH and Ganirelix, respectively. All patients were deemed to have a high risk of developing OHSS (at least 20 follicles 11mm and/or serum E2 levels of at least 3000pg/mL). There were 23.4±15.4 retrieved oocytes on average, of which 83% were mature MII oocytes. None of the patients developed OHSS-related symptoms. By segmenting IVF treatment, Devroey *et al*. (2011) proposed the “OHSS-free clinic” based on similar experiences. Pituitary down-regulation with a GnRH antagonist, ovulation induction with a GnRH agonist, and vitrification of oocytes or embryos are the key components of an OHSS-free method. The segmentation strategy for OHSS-free clinics consists of three segments. Segment A relates to the optimization of ovarian stimulation, including the use of a GnRH agonist trigger in a GnRH antagonist cycle. This is followed by segment B, which describes the most effective cryopreservation techniques for oocytes or embryo vitrification. Segment C deals with embryo replacement in a receptive, non-stimulated endometrium during a natural cycle or with artificial endometrial preparation.

However, the greatest concern associated with the use of GnRH agonists to trigger final oocyte maturation is the associated luteal phase insufficiency. During the luteal phase, LH support is essential for the maintenance of the corpus luteum. However, when a GnRH agonist is used as a trigger, the total amount of endogenous gonadotropins is significantly reduced (Gonen *et al*., 1990; Itskovitz *et al*., 1991) compared to a natural mid-cycle gonadotropin surge. A natural LH surge has three phases with a total duration of 48 h, whereas a GnRH agonist-induced LH surge has two phases with a duration of 24-36h (Humaidan *et al*., 2009). Additionally, the supraphysiological E2 levels associated with ovulation stimulation protocols exert negative feedback on the hypothalamic-pituitary axis, resulting in low endogenous LH levels during the early and middle luteal phases (Tavaniotou & Devroey, 2006; Tavaniotou *et al*., 2001). Due to luteal phase deficiency, Humaidan *et al*. (2005) concluded in a prospective randomized controlled trial that even though more MII oocytes were retrieved in patients receiving the GnRH agonist trigger, there was a significantly lower implantation rate and clinical pregnancy rate, as well as a higher rate of early pregnancy loss. In a separate clinical trial conducted by Kolibianakis *et al*. (2005), the GnRH agonist trigger yielded a significantly lower probability of ongoing pregnancy (odds ratio 0.11; 95% confidence interval [CI]: 0.02-0.52), resulting in the trial’s termination. Griesinger *et al.* (2007) observed good birth rates in frozen-thawed embryo replacement cycles, in which embryos derived from GnRH agonist-triggered cycles were transferred; the authors concluded that the likelihood of a live birth did not appear to be diminished.

Therefore, the GnRH agonist trigger is an effective strategy for enhancing MII oocyte retrieval and preventing OHSS. However, fresh embryo transfers in a GnRH agonist-triggered cycle require extensive luteal phase support to improve clinical pregnancy rates and reduce early pregnancy loss.

#### 3. Combining hCG with GnRH agonist trigger

##### 3.1 Low dose hCG supplementation after GnRH agonist: Rescue hCG

Humaidan *et al*. (2010) proposed the administration of a low dose of hCG (1,500 IU) 35 hours after inducing final oocyte maturation with a GnRH agonist trigger in fresh embryo transfers in order to reduce the recurrent pregnancy loss rate. One group received a GnRH agonist trigger (n=152) and the other group received an hCG trigger (n=150), with ovum retrieval occurring 34 hours later. A single bolus of 1,500 IU of hCG was administered intramuscularly (IM) 35 hours after GnRH agonist administration to the first group. Positive hCG per embryo transfer rate was 48% *vs*. 48%; ongoing pregnancy rates were 26% *vs*. 33%; delivery rates were 24% *vs*. 31%; and early pregnancy loss rates were 21% *vs*. 17%, respectively, without statistical differences between the two groups. Therefore, it can be concluded that a small dose of hCG administered 35 hours after GnRH agonist rescued the luteal phase from luteolysis, resulting in equivalent reproductive outcomes.

##### 3.2 GnRH agonist forty hours and standard hCG added thirty-four hours prior to ovum pickup (OPU): Double Trigger

Fruchter and colleagues presented the first case of recurrent empty follicle syndrome (EFS) treated successfully with a “double-trigger” (Beck-Fruchter *et al*., 2012). The patient underwent seven OPU procedures, with the first four yielding no oocytes and the following three yielding between one and four oocytes of poor quality. The patient received 0.1 mg of triptorelin acetate and a GnRH agonist 40 hours prior to OPU. In order to avoid unfavorable clinical pregnancy rates with a GnRH agonist trigger, 250 g of recombinant hCG was administered 34 hours prior to OPU. Aspiration resulted in 18 oocytes, of which 16 were MII oocytes. Eleven embryos developed, of which two were transferred 48 hours after OPU; nine were cryopreserved. Beginning on the day of embryo transfer, adequate luteal support with progesterone and E2 was initiated. A singleton pregnancy was achieved, and a boy weighing 2,600 g was born at 38 weeks.

Inspired by the results of the double trigger, Haas *et al*. (2019) randomized 33 patients into three groups for a pilot study on poor responders: 11 patients received hCG (6500IU) 36 hours prior to OPU (reference trigger), 10 patients received GnRH agonist trigger 36 hours prior to OPU with hCG rescue (6500IU) on day of OPU (GnRH agonist with luteal support), and 12 patients received GnRH agonist and hCG (6500IU) 40 and 34 hours prior to OPU, respectively (double trigger). In comparison to the hCG group and the GnRH agonist group, patients in the double trigger group had a significantly greater number of high-quality embryos. This result was attributed to GnRH agonists inducing an FSH surge in addition to the LH surge, which mimics the natural midcycle gonadotropin surge.

##### 3.3 Standard hCG dose in conjunction with GnRH agonist: Dual trigger

Due to the different effects of LH and hCG on the downstream signaling of the LH receptor (Riccetti *et al*., 2017; Casarini *et al*., 2012) and the more physiological gonadotropin surge produced by GnRH agonist, it is prudent to administer GnRH agonists concurrently with hCG to trigger oocyte maturation. This is referred to as a “dual-trigger.” By adding hCG to the GnRH agonist trigger, hCG not only helps to support the corpus luteum during the luteal phase, but it also assists in oocyte maturation.

Shapiro *et al.* (2008) conducted a retrospective examination of 45 antagonist down-regulated cycles, in which oocyte maturation was triggered by a dual trigger. Patients were given leuprolide acetate (4 mg) as a GnRH agonist and hCG as a trigger. Individualized hCG dosage was determined based on the patient’s profile and risk factors for OHSS. Oocytes were retrieved 34-36 hours after the trigger and inseminated using Intracytoplasmic sperm injection (ICSI); the embryos were cultured to the blastocyst stage prior to transfer. Until 8-10 weeks of gestation, all patients received luteal phase support with E2 and progesterone. In their study, each cycle triggered with the dual trigger yielded an average of 11 retrieved oocytes; none of the cycles were aborted, and all patients received an embryo transfer of either one or two blastocysts. The rate of early pregnancy loss was 17.2% (95% CI, 5.9% to 35.8%), while the rate of ongoing pregnancy was 53.3% per transfer (95% CI, 37.3% to 68.3%). No patient was diagnosed with even a mild case of OHSS, even though many of them possessed risk factors. One patient with a history of severe OHSS after receiving 5,000 IU of hCG in the previous cycle reported no OHSS symptoms after receiving the dual trigger (2,200 IU of hCG and 4 mg of leuprolide acetate). The dual trigger was considered a safe and effective strategy for oocyte maturation in high responders in terms of the ability to achieve recurrent pregnancies while reducing the risk of OHSS.

###### 3.3.1 Dual trigger in patients with a high response

Due to patient safety and ethical considerations, most studies on dual trigger in high responders with an increased risk of OHSS have been retrospective cohort studies.

Shapiro *et al.* (2011) conducted a retrospective cohort study with high responders defined as 20 follicles and/or serum E2 500 pg/mL before trigger. They compared the success rates of fresh autologous blastocyst transfers following (a) GnRH agonist administered with concomitant low-dose hCG and standard luteal support (dual trigger); (b) GnRH agonist alone and standard luteal support; and (c) GnRH agonist alone and enhanced luteal support. Their study concluded that ongoing pregnancy rates were significantly higher with the dual trigger or with enhanced luteal support (57.7% *vs*. 25% *vs*. 50%). One case of OHSS was reported in the dual trigger group and none in the GnRH agonist trigger group.

In another retrospective cohort study (O’Neill *et al.,* 2016), incidence of OHSS, total oocyte yield, and oocyte maturity were assessed in 108 high responders given a GnRH agonist trigger and 66 high responders given the dual trigger (GnRH agonist + low-dose [1000 IU] hCG trigger). In their study, the incidence of early-onset OHSS was significantly greater following dual trigger than GnRH agonist trigger (8.6 *versus* 0%). In contrast, the dual trigger was associated with a greater number of total oocytes (OR 1.27; 95% CI 1.18, 1.38) and a greater proportion of mature oocytes (OR 1.10; 95% CI 1.03, 1.17) than the GnRH agonist trigger alone. Even though the dual trigger led to a modest increase in oocyte yield, both in terms of number and maturity, the use of the dual trigger in high responders was associated with an increased risk of OHSS.

###### 3.3.2 Dual trigger in women with diminished ovarian reserve (DOR) and poor ovarian response (POR)

In 2018, the Society of Assisted Reproductive Techniques (SART) reported that 26,345 IVF cycles were performed on women between the ages of 38 and 40, with a live birth rate of 26.8%, compared to 27,624 IVF cycles performed on women between the ages of 35 and 37, with a live birth rate of 40%. Improving reproductive outcomes in women with advanced age and DOR is one of the greatest challenges in contemporary ART practice.

The first successful attempt to define POR was produced by the European Society of Human Reproduction and Embryology (ESHRE) consensus on the definition of poor response to ovarian stimulation: the “Bologna criteria” (Ferraretti *et al*., 2011). The more recent POSEIDON (Patient-Oriented Strategy Encompassing IndividualizeD Oocyte Number) criteria provides a more specific definition of poor prognosis patients and categorizes patients into four different groups with varying degrees of poor prognosis (Poseidon Group, 2016).

A recent randomized controlled trial in poor responders has provided some evidence that dual trigger is associated with better IVF outcomes than the single hCG trigger (Maged *et al*., 2021). A total of 160 women who met the Bologna criteria for POR (Ferraretti *et al*., 2011) were randomly divided into two groups: group I received 10,000 IU of hCG plus 0.2 mg of triptorelin and group II received 10,000 IU of hCG alone for inducing ovulation. The primary outcome measure was the number of oocytes retrieved. The number of oocytes in metaphase II, cancellation rate, the number of embryos obtained, and chemical and clinical pregnancy rates were secondary outcomes. Dual trigger was associated with an increase in the number of retrieved oocytes (5.3±1.9 *vs*. 4.5±2.4, *p*=0.014), metaphase II oocytes (3.8±1.4 *vs*. 3.1±1.7, *p*=0.004), total and grade 1 embryos (2.7±1.1 and 2.3±1.0 *vs*. 1.9±1.2 and 1.1±0.2, *p*=0.001 and 0.021, respectively)

Previously, a retrospective analysis of 427 GnRH-antagonist downregulated IVF cycles with fresh embryo transfers had demonstrated improved reproductive outcomes in DOR patients given the dual trigger (Lin *et al*., 2019). The criteria for DOR were AFC 5 and serum AMH 1.1 ng/mL. The study group (n=297) was triggered with 0.2 mg of triptorelin plus 6500 IU of recombinant hCG, whereas the control group (n=130) was triggered with 6500 IU of recombinant hCG. They observed that oocyte fertilization rate (73.1% *vs*. 58.6%), clinical pregnancy rate (33.0% *vs*. 20.7%), and live birth rate (26.9% *vs*. 14.5%) were all higher in the dual trigger group than in the hCG trigger group. In the dual trigger group, the abortion rate (17.4% *vs*. 37.0%) and embryo transfer cancellation rate (6.1% *vs*. 15.0%) were also significantly lower than in the control group. The authors concluded that in women with DOR, the dual trigger could significantly increase fertilization, clinical pregnancy, and live birth rates.

Chern *et al*. (2020) investigated 384 GnRH antagonist down-regulated IVF cycles meeting the POSEIDON group 4 criteria. A total of 194 cycles were performed with the dual-trigger (r-hCG 250 mcg plus leuprolide acetate 2 mg) for final oocyte maturation in the study group. The control group consisted of 114 cycles triggered with 250 mcg of r-hCG. In this study, both the number of embryos transferred (2.1±1.0 *vs*. 1.4±0.8, *p*<0.001) and the proportion of high-quality embryos transferred (62.5 *vs*. 23.9%, *p*<0.001) were significantly greater in the dual trigger group than in the hCG group. In terms of implantation rate (14.4±30.0% *vs*. 5.4±18.8%, *p*=0.004), clinical pregnancy rate (23.1% *vs*. 8.7%, *p*=0.004), and live birth rate (17.5% *vs*. 5.4%, *p*=0.006), the dual-trigger group performed better. In addition, the study demonstrated that AMH is a positive independent factor influencing clinical pregnancy rates (OR = 7.20, 95% CI = 1.33-39.61, *p*=0.023). Thus, the authors concluded that the dual trigger was superior to hCG trigger for women meeting the POSEIDON group 4 criteria.

Young women with DOR who wish to cryopreserve oocytes for fertility preservation benefit most from the dual trigger. A retrospective analysis (Kim *et al.,* 2020) of 76 cycles for elective oocyte cryopreservation with the intent of fertility preservation in women younger than 35 years old with AMH levels <1.2 ng/ml studied two intervention groups: a dual trigger (0.2 mg decapeptyl and 250 mcg of r-hCG, n=40) and a trigger of 250 mcg of r-hCG alone (n=36). In both groups, the total number of retrieved oocytes was comparable (5.3±3.5 *vs*. 5.0±2.7, *p*=0.655). However, significantly more mature oocytes were recovered in the dual trigger group compared with the hCG group (3.7±2.7 *vs*. 2.3±1.7, respectively; *p*=0.010). Consequently, women who undergo elective oocyte cryopreservation for fertility preservation are more likely to experience favorable results from the dual trigger.

###### 3.3.3. Dual triggering in normal responders (Table 1)

In a prospective randomized controlled trial (Decleer *et al*., 2014), 120 normal responders undergoing ICSI were randomized into two groups. The first group (n=59) was administered 5,000 IU of hCG, while the second group (n=61) received a combination of GnRH agonist (0.2 mg) and 5,000 IU of hCG. In the hCG-triggered group, the average number of MII oocytes was 9.2 compared to 10.3 in the dual-trigger group. The number of patients who received at least one embryo of excellent quality was significantly greater (*p*=0.001) in the group with dual triggers (45 of 61 patients or 73.8%) than in the group with hCG triggering alone (28 of 59 patients or 47.5%).

**Table 1:** Comparison of study design, sample size, triggers and ART procedure in different studies.

Author	Study Design	Size			Trigger		ART Procedure
		Total Participants	Intervention Group	Reference Standard group	Intervention group	Reference Standard Group	ART Procedure
Schachter *et al*., 2008	Prospective RCT	221	105	106	Triptorelin 0.2 mg + hCG 5,000 IU	hCG 5,000 IU	IVF and IVF-ICSI
Lin *et al*., 2013	Retrospective Cohort	376 (378 cycles)	191	187	Triptorelin 0.2 mg + rhCG 250 µg	rhCG 250 µg	IVF and IVF-ICSI
Decleer *et al*., 2014	Prospective RCT	120	61	59	Triptorelin 0.2 mg + hCG 5,000 IU	hCG 5,000 IU	IVF-ICSI
Mahajan *et al*., 2016	Prospective RCT	76	38	38	Leuprolide 1 mg + hCG 5,000 IU	hCG 10,000 IU	IVF-ICSI
Seval *et al*., 2016	Retrospective Case Control	156	84	72	Leuprolide acetate 0.5 mg + rhCG 250 µg	rhCG 250 µg	IVF and IVF-ICSI
Alleyassin *et al*., 2018	Prospective RCT	126	63	63	Triptorelin 0.2 mg + hCG 5,000IU	hCG 10,000 IU	IVF-ICSI
Ali *et al*., 2020	Prospective RCT	160	80	80	Leuprolide 1 mg + rhCG 250µg	rhCG 250 µg	IVF-ICSI
Haas *et al*., 2020	Prospective RCT	155	77	78	Buserelin 0.5 mg + 10,000 IU hCG	hCG 10,000 IU + Placebo (normal saline)	IVF and IVF-ICSI

In a prospective randomized study conducted in an Asian population (Mahajan *et al*., 2016), 76 patients were randomly assigned to receive either 10,000 IU of hCG (n=38) or dual trigger with 1 mg of leuprolide acetate and 5,000 IU of hCG (n=38). Excluding high responders at risk for OHSS, the study included women with AMH <4 ng/ml and AFC/ovary <12. The study found no statistically significant differences between the dual group and the hCG group in terms of the number of oocytes retrieved (10.0±5.6 *vs*. 8.7±5.0; *p*=0.2816), the number of mature oocytes recovered (8.4±5.0 *vs*. 7.2±4.0; *p*=0.2588), the fertilization rate (5.9±4.2 *vs*. 5.6±3.3; *p*=0.7390). A subgroup analysis of women with AMH 1,4 ng/ml revealed no significant difference between the two groups in terms of the number of viable embryos.

Eight randomized controlled trials (RCT) were included in the final analysis of a recent systematic review and meta-analysis (Hu *et al*., 2021). Dual trigger therapy was associated with a significantly higher live birth rate (LBR) per initiated cycle compared to hCG trigger therapy (RR = 1.37 [1.07, 1.80], I^2^ = 0%, moderate evidence). The authors concluded that this effect may be mediated by an increase in the number and quality of oocytes and embryos observed with the dual trigger.

Ding *et al*. (2017) had previously conducted a systematic review and meta-analysis that included 4 RCTs with a total of 523 women comparing IVF outcomes between dual trigger and hCG trigger in GnRH antagonist down-regulated cycles. The results of this meta-analysis indicated that the dual trigger group had a significantly higher pregnancy rate than the hCG-only trigger group (RR 1.55; 95% CI 1.17-2.06). There were no significant differences between the two groups in terms of the number of oocytes retrieved, the number of mature oocytes retrieved, the number of fertilized oocytes, the number of high-quality embryos, or the implantation rates.

More recently, Hass *et al*. (2020) conducted a double-blind prospective randomized controlled trial to compare the efficacy of dual trigger and hCG trigger in normal responders After excluding patients at high risk of OHSS (E2 levels >15,000 pmol/L or AFC >20), patients with BMI >35, patients with moderate to severe endometriosis, and patients with low ovarian reserve, 155 patients were randomized into two groups: (i) hCG group (n=78): patients were triggered for final follicular maturation with hCG (Pregnyl 10 000 IU) and placebo (normal saline); and (ii) dual trigger group (n=77): patients were triggered with a GnRH agonist (Suprefact 0.5 mg) and hCG (Pregnyl 10,000 IU) 36 h prior to oocyte aspiration. The number of eggs retrieved (11.1 *vs*. 13.4, *p*=0.002), MII oocytes (8.6 *vs*. 10.4, *p*=0.009), the total number of blastocysts (2.9 *vs*. 3.9, *p*=0.01), and the percentage of high-quality blastocysts transferred (44.7% *vs*. 64.9%; *p*=0.003) were significantly greater in the dual trigger group than in the hCG group. Clinical pregnancy (24.3% *vs*. 46.1%, OR 2.65 [1.43-1.93], *p*=0.009) and live birth rates per transfer (22% *vs*. 36.5%, OR 1.98 [1.03-3.75], *p*=0.03) were also significantly higher in the dual trigger group compared to the hCG group.

The ESHRE Guideline Group on Ovarian Stimulation (2020) do not recommend the addition of a GnRH agonist to hCG as a dual trigger for the maturation of mature oocytes in predicted normal responders. However, the strength of the supporting evidence is low, and the quality of the available meta-analyses has been rated as poor. The authors have emphasized the need for additional evaluation with well-designed RCTs in order to reach a more definitive conclusion.

## SUMMARY

In conclusion, this review found favorable outcomes associated with dual trigger in most situations. Even though current guidelines do not recommend the routine use of the dual trigger in normal responders, recent studies have shown benefits associated with the dual trigger in terms of both embryological and reproductive outcomes, which can be indirectly translated into better cumulative births. Thus, maybe it is time for us to reconsider the dual trigger as the trigger of choice in all patients undergoing antagonist cycle IVF, with the exception of individuals with polycystic ovary syndrome (PCOS). Future research should be directed at planning adequately powered, large multicentric clinical trials which aim to compare outcomes of both fresh and frozen cycles, in terms of cumulative pregnancy rate. There is a need to understand the molecular mechanisms involved with the dual trigger and to standardize the dosage and type of GnRH agonist and hCG in the dual trigger combination, so as to achieve maximal reproductive outcomes, that are cost-effective and safe for patients.
